# The Association Between Dietary Vitamin C and Sleep Disorders: A Cohort Study Based on UK Biobank

**DOI:** 10.3390/nu16213661

**Published:** 2024-10-28

**Authors:** Qiuge Zhang, Xueting Qi, Zhaoguo Wang, Dongfeng Zhang, Tong Wang

**Affiliations:** 1 Department of Epidemiology and Health Statistics, Public Health College, Qingdao University, Qingdao 266021, China; zhangqiuge@qdu.edu.cn (Q.Z.); qixueting@qdu.edu.cn (X.Q.); zhangdongfeng@qdu.edu.cn (D.Z.); 2 Qingdao Centers for Disease Control and Prevention, Qingdao 266021, China; cdcwzg@qd.shandong.cn

**Keywords:** sleep disorder, diet, vitamin c, UK Biobank

## Abstract

**Objective:** Approximately 30% of adults globally suffer from sleep disorders. However, there are few longitudinal studies on the association between dietary vitamin C and sleep disorders. This study aimed to investigate the association between dietary vitamin C intake and various types of sleep disorders, including sleep apnea and insomnia. **Methods:** We enrolled 68,221 participants from the UK Biobank. Dietary vitamin C intake was assessed using a 24 h dietary recall questionnaire. We employed a Cox regression model to assess the association between dietary vitamin C and sleep disorders and used restricted cubic spline models to investigate potential nonlinear relationships. Stratified and sensitivity analyses were also conducted to validate the findings. **Results:** The results indicated that vitamin C intake at the Q4 level (132.88–191.51 mg/d) was significantly associated with a reduced risk of sleep disorders, with an HR (95% CI) of 0.78 (0.65–0.94), and against sleep apnea, with an HR (95% CI) of 0.75 (0.62–0.92). The protective effect persisted significant in stratified analyses of men and those aged ≤ 60 years; the results were consistent in the sensitivity analyses. **Conclusions:** Our findings suggest that sufficient dietary vitamin C intake may help to prevent sleep disorders.

## 1. Introduction

Sleep disorders comprise a group of conditions that disrupt normal sleep patterns, including insomnia, sleep apnea, sleep deprivation, sleep–wake rhythm disorders, and other sleep disorders (including REM behavior disorder, restless leg syndrome, etc.) [1]. As a global public health issue, sleep disorders affect approximately 30% of adults worldwide [2,3]. In addition to directly impairing quality of life [4], sleep disorders contribute to various physical and psychological complications, including diabetes [5,6,7], cardiovascular disorders [8,9], cognitive decline [10,11], and stroke [12]. The coexistence of sleep disorders with multiple chronic diseases significantly impacts individual health, public health systems, and socioeconomic factors [13,14,15]. Previous studies have shown that consuming antioxidant-rich foods can significantly improve sleep disorders [16].

Vitamin C is a potent antioxidant [17] that reduces oxidative stress by absorbing and neutralizing free radicals produced by the body [18,19]. Several cross-sectional studies have elevated the effect of vitamin C on sleep disorders. A study in Japan found an association between dietary vitamin C and insomnia [20]. Additionally, another study has identified a correlation between serum vitamin C and sleep apnea [21]. Mhaidat N.M. et al. found that dietary vitamin C reduced sleep deprivation-induced memory impairment [22]. Moreover, dietary vitamin C intake prevents sleep–wake disorders [23]. The antioxidant vitamin C can effectively reduce the incidence of restless leg syndrome [24]. Another study utilizing NHANES data identified a significant negative association between serum vitamin C levels and sleep disorders [25].

In summary, these articles are cross-sectional studies; no longitudinal studies have yet addressed this topic. Moreover, most cross-sectional studies had relatively small sample sizes and lacked a clear definition of sleep disorders, and there were no articles analyzing sleep disorders as defined by ICD10. Therefore, we conducted a longitudinal analysis using UK biobank data to investigate the causal association between dietary vitamin C intake and sleep disorders or specific sleep disorders.

## 2. Materials and Methods

### 2.1. Study Population

**Figure 1:**
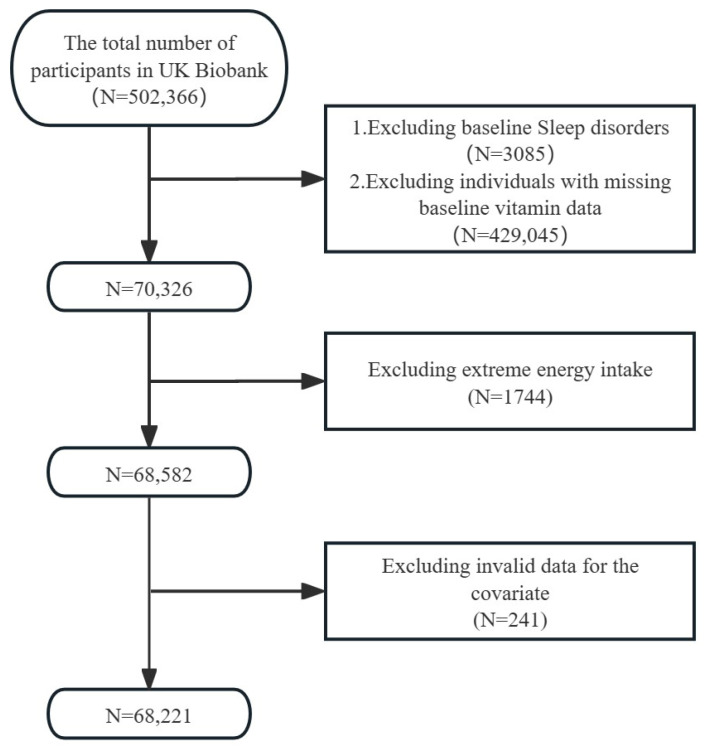
Flowchart of study participants.

This study utilized data from the UK Biobank (https://www.ukbiobank.ac.uk/, accessed on 25 October 2022), a comprehensive, population-based, prospective study. Evaluation visits included electronic signature consent forms, self-administered touchscreen questionnaires, brief computer-assisted interviews, the measurement of physical and functional capabilities, and the collection of blood, urine, and saliva samples [26]. The UK Biobank data were collected during the initial survey conducted from 2006 to 2010, with ongoing follow-ups to the present. Demographic information and health behaviors were derived from this period (2006–2010), while the 24 h dietary data and data on the use of vitamin C supplements were obtained from the first survey conducted in 2009–2010. Sleep disorders, along with diabetes, hypertension, stroke, and cancer, were defined according to International Classification of Diseases 10 (ICD10). The onset of sleep disorders was based on the time when the participant first came to participate in the survey (during 2006–2010) as a start time, with event endpoints, as follows at: (1) time of first recording of the patient’s illness; (2) deathtime; and (3) the cut-off time (30 September 2023) for our data download. The North West Multi-centre Research Ethics Committee (MREC) granted ethical approval after all participants gave their informed consent. The data from this study were approved, with the approval number 95715. The inclusion criteria for this study were as follows: (1) absence of sleep disorders at baseline; and (2) complete dietary vitamin C data at baseline. The exclusion criteria were as follows: (1) extreme energy intake; and (2) incomplete covariate data. Ultimately, a total of 68,221 participants were included in the analysis. Figure 1 shows a flowchart of the screening process for the selection of eligible participants.

### 2.2. Assessment of Sleep Disorder

Our primary outcome variable, sleep disorders, was identified using ICD10 codes obtained through coordination with national registries. Specifically, instances of sleep disorders were classified using ICD-10 codes G47.0-9 (Supplementary Table S1). Onset time was based on when participants first participated in the survey (between 2006–2010) as a start time, with the event endpoints as follows: (1) when the patient’s illness was first recorded; (2) time of death; and (3) up to the time of our data download (30 September 2023).

### 2.3. Assessment of Dietary Vitamin C Intake

The UK Biobank’s Internet-based 24 h dietary questionnaire (Oxford WebQ) was the first survey conducted from 2009–2010. We conducted a correlation analysis on participants who completed at least two vitamin C surveys (N = 49,117). The results showed a significant correlation of vitamin C intake in the two surveys (*r* = 0.44, *p* < 0.05), suggesting some stability and representativeness of the survey method. With the maximum number of persons at the baseline, we used baseline data to assess dietary vitamin C intake. Participants were invited to complete multiple dietary review questionnaires to obtain dietary information at different time points, reducing individual differences in dietary intake. To ensure the accuracy of the data, the UK Biobank conducts quality control of dietary data, excluding unreasonable dietary reports or missing data. The data were then categorized into the following five groups: Q1 (<51.27 mg/d); Q2 (51.27–90.44 mg/d); Q3 (90.44–132.88 mg/d); Q4 (132.88–191.51 mg/d); and Q5 (>191.51 mg/d), based on quintiles.

### 2.4. Assessment of Covariates

Based on previous studies in the literature regarding diet intake and sleep [27,28,29], we included a series of covariates in our study. The demographic variables included gender, age, education level, household income, Townsend Deprivation Index (TDI), and ethnicity. Additionally, we incorporated several health behavior variables such as body mass index (BMI), physical activity, smoking status, alcohol drinker status, vitamin C supplements, and total energy intake. We also included comorbidities such as hypertension [30], diabetes [5], stroke [31], and cancer [32].

### 2.5. Statistical Analysis

We compared the characteristics and dietary vitamin C intake of individuals with and without sleep disorders. The normality of continuous variables was assessed using the Kolmogorov–Smirnov test. Normally distributed continuous variables were expressed as mean ± standard deviation (SD) and compared using Student’s *t* test, while non-normally distributed variables were presented as median and interquartile range (IQR) and analyzed using the Mann–Whitney U test. Categorical variables were presented as frequencies and percentages and analyzed using the chi-squared (χ^2^) test.

To estimate the association between dietary vitamin C intake and the risk of sleep disorders, we employed a Cox regression model to estimate the hazard ratio (HR) and its 95% confidence interval (CI). We further explored the effects of dietary vitamin C on sleep apnea, insomnia, and other sleep disorders. Survival time was defined as the interval from the beginning of follow-up to the occurrence of the endpoint event. For participants who did not experience the endpoint event, survival time was calculated up to their death or the end of the follow-up period. The lowest vitamin C intake group (Q1) was used as the reference group. Multicollinearity was assessed using the variance inflation factor (VIF), and no signs of multicollinearity were detected in any of the models. We constructed three models:

Model 1 was the crude model without any confounders;

Model 2 adjusted for age and gender (male, female);

Model 3 included additional variables, as follows: race (white, other) education level (college or university degree = 1, A levels/AS levels or equivalent = 2, O levels/GCSEs or equivalent = 3, CSEs or equivalent = 4, NVQ or HND or HNC or equivalent = 5, other professional qualifications, e.g., nursing, teaching = 6, other = 0); household income (less than 18,000 = 1, 18,000 to 30,999 = 2, 31,000 to 51,999 = 3, 52,000 to 100,000 = 4, greater than 100,000 = 5, other = 0); TDI; BMI; physical activity; smoking status (never = 0, previous = 1, current = 2), alcohol drinker status (never = 0, previous = 1, current = 2); total energy intake; vitamin C supplements (yes = 1, no = 0); hypertension; diabetes; stroke; and malignant neoplasms. Due to dietary vitamin C intake having potential anti-inflammatory properties, we used c-reactive protein as a mediator to test whether it mediates the effect of dietary vitamin C and sleep disorders. Analyses were performed using the “mediation” package in R 4.2.3. The bootstrap method was performed for 1000 iterations and adjusted using model 3.

Given the varying prevalence of sleep disorders across genders and ages, we conducted stratified analyses based on these variables. Furthermore, considering previous research indicating that sleep-related breathing disorders, insomnia, and insufficient sleep can contribute to elevated blood pressure, we specifically examined the effect of dietary vitamin C intake on sleep disorders among individuals with and without hypertension.

We used a restricted cubic spline model with four knots to assess the dose–response relationship between dietary vitamin C intake and the risk of sleep disorders, assessing the effects across various dosage levels.

In our sensitivity analyses, which accounted for potential delays in disease onset, we excluded subjects who developed sleep disorders within two years of the investigation, and further analyzed associations between dietary vitamin C and sleep disorders, as well as sleep apnea, insomnia, and other types of sleep disorders. Results were considered statistically significant when the *p*-value on both sides was less than 0.05.

## 3. Results

This survey included a total of 68,221 participants who met the inclusion criteria. The average age was 56.24 ± 8.14 years. Table 1 presents a comparison of characteristics between participants with and without sleep disorders. Those with sleep disorders were generally older, more likely to be male, and had a higher BMI. They also smoked more frequently, consumed more total energy, and had higher rates of hypertension, stroke, diabetes, and malignant neoplasms. Additionally, they tended to have lower family incomes, education levels, Townsend Deprivation Index scores, MET scores, and vitamin C intake.

**Figure 2:**
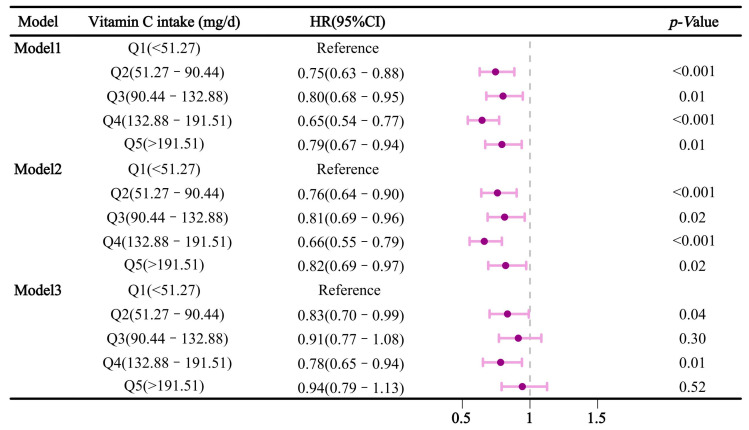
Association between dietary vitamin C intake and sleep disorders.

Figure 2 illustrates the relationship between dietary vitamin C intake and sleep disorders. In Model 1, increased vitamin C intake was significantly associated with a reduced risk of sleep disorders (*p* < 0.05). Compared to the lowest intake group (Q1), the hazard ratios (HR) and 95% confidence intervals (CI) for Q2, Q3, Q4, and Q5 were 0.75 (0.63–0.88), 0.80 (0.68–0.95), 0.65 (0.54–0.77), and 0.79 (0.67–0.94), respectively. In Model 2, these associations persisted with HRs (95% CI) of 0.76 (0.64–0.90), 0.81 (0.69–0.96), 0.66 (0.55–0.79), and 0.82 (0.69–0.97) for Q2, Q3, Q4, and Q5, respectively. However, in Model 3, Q2 and Q4 showed significant protective effects against sleep disorders, with HRs (95% CI) of 0.83 (0.70–0.99) and 0.78 (0.65–0.94), compared to Q1.

**Figure 3:**
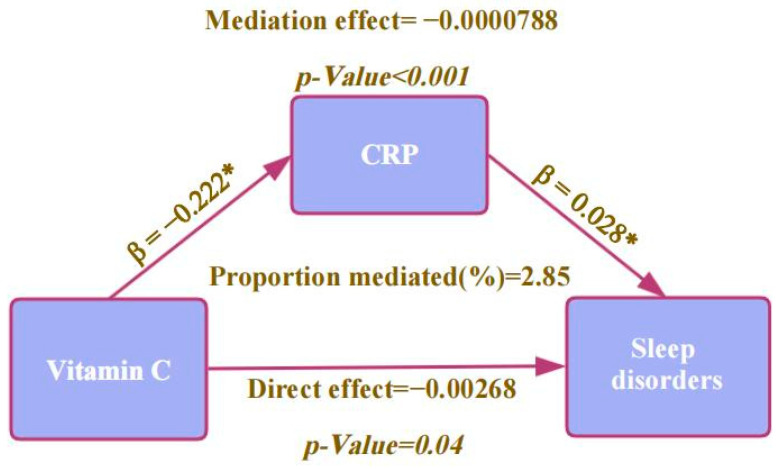
Mediation analysis pathway diagram for the relationship between vitamin C, CRP, and sleep disorders. * *p* < 0.05.

Based on previous studies showing that Vitamin C reduced the inflammatory response, which has been associated with sleep disorders, we chose C-reactive protein (CRP), a classic indicator of the inflammatory response, to conduct the mediation analysis. The results, as illustrated in Figure 3, indicate that CRP plays a mediating role in the effect of dietary vitamin C on sleep disorders, with a mediation proportion of 2.85% (*p* < 0.05).

**Figure 4:**
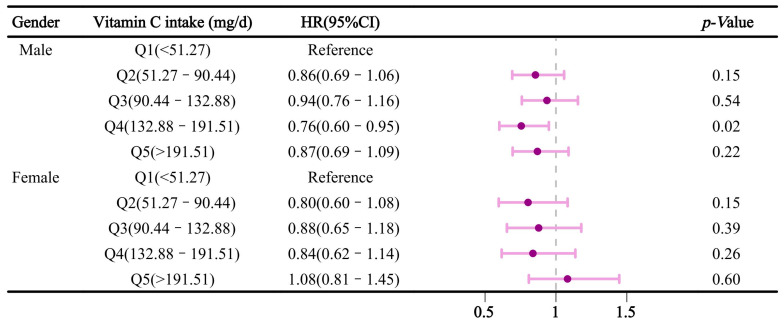
Association between dietary vitamin C intake and sleep disorders after gender stratification.

**Figure 5:**
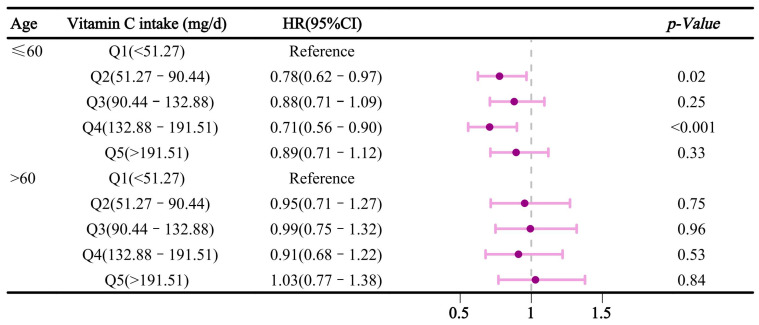
Association between dietary vitamin C intake and sleep disorders after age stratification.

**Figure 6:**
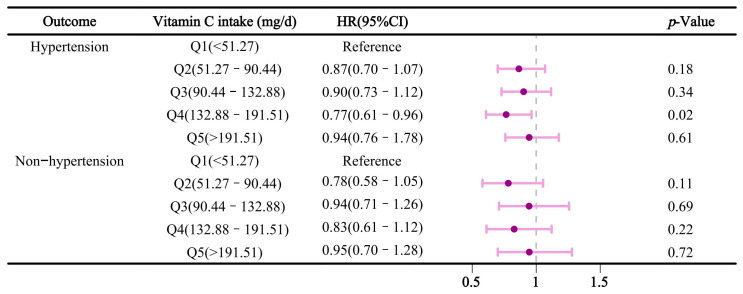
Association between dietary vitamin C intake and sleep disorders stratified by hypertension status.

We conducted a stratified analysis by gender and age. Figure 4 illustrates the relationship between vitamin C intake and sleep disorders after stratification by gender. In the multivariable-adjusted model, compared to Q1, dietary vitamin C intake in Q4 was inversely associated with sleep disorders in males, with an HR (95% CI) of 0.76 (0.60–0.95). However, this association was not significant in females. Additionally, in the age-stratified analysis (Figure 5), dietary vitamin C intake in participants aged ≤ 60 was negatively associated with sleep disorders in Q2 and Q4, with HRs (95% CI) of 0.78 (0.62–0.97) and 0.71 (0.56–0.90), respectively. Figure 6 reveals stratified results by hypertension, indicating that dietary vitamin C intake in Q4 had a significant effect on sleep disorders in hypertensive individuals, with an HR and 95% confidence interval of 0.77 (0.61–0.96). Moreover, subsequent analyses of vitamin C and sleep apnea revealed consistent results with the primary findings (Supplementary Tables S2–S5). In Model 3, a protective effect of dietary vitamin C against sleep apnea was observed in Q4, with HRs (95% CI) of 0.75 (0.62–0.92), compared to those of Q1. Nevertheless, no significant effect of dietary vitamin C was found on insomnia or other types of sleep disorders (Supplementary Tables S6–S12).

**Figure 7:**
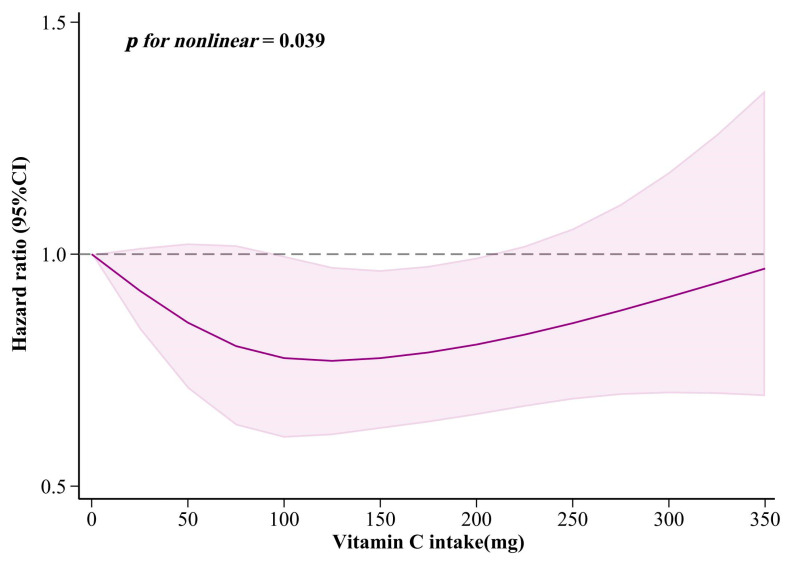
The results of dose–response relationship between dietary vitamin C intake and sleep disorders. The solid purple line represents the HR value and the shaded area represents the confidence interval.

The dose–response relationship is illustrated in Figure 7. There was a nonlinear association between dietary vitamin C intake and sleep disorders (*p* for nonlinear = 0.039). Specifically, when vitamin C intake ranged between approximately 125 and 200 mg/day, the risk of sleep disorders was notably reduced. Furthermore, Supplementary Figures S1–S3 show the results of the dose–response relationship in males, participants aged ≤ 60, and individuals with hypertension.

Table 2 reveals the sensitivity analysis results. After excluding participants who developed sleep disorders within two years, the findings remained consistent with the main findings. Compared to Q1, dietary vitamin C intake in Q4 still showed a protective effect against sleep disorders, with an HR of 0.80 (95% CI: 0.66–0.97). Similarly, for sleep apnea, the HR was 0.77 (95% CI: 0.62–0.95).

## 4. Discussion

Our results showed that higher dietary vitamin C intake was associated with a reduced risk of sleep disorders, especially in males, individuals aged 60 years or younger, and those with hypertension. This protective effect persisted in patients with sleep apnea but was not observed in cases of insomnia or other sleep disorders. C-reactive protein significantly mediated the effect of dietary vitamin C on sleep disorders. Additionally, in analyses excluding participants who developed sleep disorders within two years, the results remained robust. These results suggest that vitamin C plays a crucial role in mitigating the risk of sleep disorders.

Previous research in rats has demonstrated that vitamin C reduces oxidative and carbonyl stress in obstructive sleep apnea and prevents metabolic, hormonal, and lipid peroxidation issues resulting from sleep deprivation [33]. In the general population, a daily intake of approximately 110 mg is recommended to maintain adequate circulating vitamin C concentrations of 50 µmol/L [34], suggesting that serum vitamin C levels reflect dietary intake. This is consistent with the protective effect of dietary vitamin C on sleep disorders observed in our study.

Previous studies have identified strong associations with age stratification in individuals under 65 years, closely aligning with our findings in those under 60 years. This correlation may stem from age-related changes in vitamin absorption due to altered dietary needs and declining digestive system function [35,36]. Our study showed a significant effect of vitamin C intake on sleep disorders in males, but not in females. While we observed no link between vitamin C and insomnia, contrasting findings from another study did report an association between dietary vitamin C intake and sleeplessness [20]. The cross-sectional study methodology, sample size selection, and regional variances could all be contributing factors to these discrepancies.

Vitamin C may be associated with sleep disorders through the following mechanisms. Vitamin C may reduce the incidence of sleep disorders through its anti-inflammatory and antioxidant properties [16,37]. The mediated analysis of our study showed that higher dietary vitamin C was related to elevated CRP, which negatively affected sleep quality [38]. CRP may partially mediate the association between dietary vitamin C and sleep disorders. These results are consistent with our analysis. Studies revealed that ascorbic acid can enhance vascular function in sleep apnea patients with endothelial dysfunction by interacting with tetrahydrobiopterin (BH4) and maintaining its reduced state, as well as through endothelial nitric oxide synthase [39]. Additionally, adrenocorticotropic hormone increased intravenous vitamin C concentrations [40], while the synthetic hexapeptide growth hormone-releasing peptide (GHRP-6) improved sleep quality by stimulating the secretion of hormones from the hypothalamic–pituitary–adrenal (HPA) axis [41].

There are several strengths in our study. First, this large cohort size dietary vitamin C intake survey was evenly distributed throughout the year; correlation analyses between dietary vitamin C intake at baseline and intake at follow-up showed a high correlation, suggesting that our results are reliable. Second, by utilizing ICD-10 criteria for diagnosing sleep disorders, we ensured consistency in identification, reducing misunderstandings and errors and improving data accuracy and comparability. Third, we performed stratified analyses by age, gender, and hypertension status to control for confounding variables, thus enhancing the precision and interpretability of our findings, and more accurately assessing the independent effects of vitamin C on sleep disorders. Fourth, in the sensitivity analysis, we excluded individuals who developed conditions within two years of follow-up, which mitigated the impact of reverse causality and enabled a more precise evaluation of the long-term relationship between vitamin C and sleep disorders. Moreover, we used restricted cubic splines to analyze the relationship, quantify the association between vitamin C intake and sleep disorders, reveal the dose–response relationship, and help to determine the optimal dose of vitamin C, providing scientific evidence for health interventions. Finally, our multivariable analysis adjusted for dietary energy intake and other disease-related confounders, minimizing their impact and enhancing the accuracy of our findings regarding the relationship between vitamin C and sleep disorders.

Our study also has several limitations. First, we used 24 h dietary recalls, which might introduce memory biases and potentially affect the accuracy of our findings; more research is needed to confirm the results of this study. Second, we did not consider the effect of cooking on vitamin C levels. Additionally, the UK Biobank includes half a million participants from the UK, which may introduce geographical limitations. Finally, there are no interventional studies related to vitamin C; as a commonly used supplement, vitamin C can be considered for further analysis in the future.

## 5. Conclusions

Adequate vitamin C intake reduced the risk of developing sleep disorders. This association remained consistent among male participants and those ≤60 years old, with similar results observed for sleep apnea. Therefore, it is important to consume sufficient dietary vitamin C and maintain a nutritional balance in one’s daily diet.

## Figures and Tables

**Table 1:** Baseline characteristics of participants based on sleep disorders in the UK Biobank 2006–2010 (N = 68,211).

	Non-Sleep Disorder (66,998)	Sleep Disorder (1223)	*p*-Value
Age, y, mean ± SD ^a^	56.23 ± 8.15	56.93 ± 7.84	0.005
Sex (%) ^b^			
Female	37,646 (56.2)	441 (36.06)	
Male	29,352 (43.82)	782 (63.94)	
BMI, kg/m^2^, mean ± SD ^a^	27.02 ± 4.61	31.96 ± 6.93	<0.001
TDI, mean ± SD ^a^	−1.33 ± 2.81	−0.54 ± 3.08	<0.001
Average total household income before tax (%) ^b^			<0.001
Less than 18,000	10,473 (15.64)	289 (23.63)	
18,000 to 30,999	14,756 (22.03)	285 (23.3)	
31,000 to 51,999	16,342 (24.40)	263 (21.5)	
52,000 to 100,000	13,540 (20.24)	211 (17.25)	
Greater than 100,000	4186 (6.25)	53 (4.33)	
Other	7701 (11.56)	122 (9.98)	
Education (%) ^b^			<0.001
College or University degree	26,093 (38.98)	391 (31.97)	
A levels/AS levels or equivalent	8585 (12.82)	126 (10.3)	
O levels/GCSEs or equivalent	14,714 (21.98)	285 (23.3)	
CSEs or equivalent	3441 (5.14)	77 (6.30)	
NVQ or HND or HNC or equivalent	3730 (5.57)	100 (8.18)	
Other professional qualifications, e.g., nursing, teaching	3326 (4.96)	66 (5.4)	
Other	7109 (10.67)	178 (14.55)	
Race (%) ^b^			0.001
White	62,776 (93.70)	1118 (91.41)	
Other	4222 (6.30)	105 (8.59)	
Summed MET minutes per week for all activity, media (IQR) ^a^	1841 (2701)	1354 (2564)	<0.001
Smoking status (%) ^b^			<0.001
Never	38,144 (56.94)	565 (46.2)	
Previous	23,311 (34.81)	522 (42.68)	
Current	5543 (8.27)	136 (11.12)	
Alcohol drinker status (%) ^b^			<0.001
Never	2504 (3.74)	62 (5.07)	
Previous	2236 (3.34)	77 (6.3)	
Current	62,258 (92.92)	1084 (88.63)	
Total energy intake, kcal/day, mean ± SD ^a^	2024.65 ± 614.30	2087.27 ± 658.20	<0.001
Vitamin C intake, mg/d, media (IQR) ^a^	111.07 (112.61)	103.93 (119.66)	0.002
Vitamin C supplements (%) ^b^	6393 (9.54)	105 (8.59)	0.280
Stroke (%) ^b^	165 (0.25)	5 (0.41)	0.401
Diabetes (%) ^b^	4898 (7.31)	326 (26.66)	<0.001
Hypertension (%) ^b^	19,215 (28.68)	792 (64.75)	<0.001
Malignant neoplasms (%) ^b^	12,379 (18.48)	288 (23.55)	<0.001

SD, standard deviation; IQR, interquartile range; BMI, body mass index; TDI, Townsend Deprivation Index. ^a^ *p*-value was tested by Mann–Whitney U test; ^b^ *p*-value was tested by the chi-squared test.

**Table 2:** The relationship between dietary vitamin C intake and the incidence of sleep disorders, sleep apnea, insomnia, and other types of sleep disorders in participants after two years.

Outcome	Vitamin C Intake (mg/d)	HR (95%CI)	*p*-Value
Sleep disorders	Q1 (<51.27)	Reference	
	Q2 (51.27–90.44)	0.84 (0.70–1.00)	0.055
	Q3 (90.44–132.88)	0.90 (0.75–1.08)	0.253
	Q4 (132.88–191.51)	0.80 (0.66–0.97)	0.024
	Q5 (>191.51)	0.90 (0.75–1.09)	0.274
Sleep apnea	Q1 (<51.27)	Reference	
	Q2 (51.27–90.44)	0.83 (0.69–1.02)	0.07
	Q3 (90.44–132.88)	0.94 (0.77–1.13)	0.499
	Q4 (132.88–191.51)	0.77 (0.62–0.95)	0.013
	Q5 (>191.51)	0.88 (0.72–1.08)	0.231
Insomnia	Q1 (<51.27)	Reference	
	Q2 (51.27–90.44)	1.02 (0.56–1.84)	0.961
	Q3 (90.44–132.88)	0.73 (0.38–1.39)	0.337
	Q4 (132.88–191.51)	0.99 (0.54–1.81)	0.97
	Q5 (>191.51)	0.87 (0.46–1.63)	0.664
Other types of sleep disorders	Q1 (<51.27)	Reference	
	Q2 (51.27–90.44)	0.84 (0.45–1.55)	0.577
	Q3 (90.44–132.88)	0.62 (0.31–1.23)	0.17
	Q4 (132.88–191.51)	1.07 (0.59–1.96)	0.82
	Q5 (>191.51)	1.15 (0.63–2.10)	0.657

Covariates adjusted according to Model 3.

## Data Availability

Data are contained within the article. The original contributions presented in the study are included in the article/Supplementary Materials, further inquiries can be directed to the corresponding author.

## Supplementary Materials

The following supporting information can be downloaded at: https://www.mdpi.com/article/10.3390/nu16213661/s1, Table S1: Codes and names of sleep disorders defined according to ICD10. Table S2: Association between dietary vitamin C intake and sleep apnea. Table S3: Association between dietary vitamin C intake and sleep apnea after gender stratification. Table S4: Association between dietary vitamin C intake and sleep apnea after age stratification. Table S5: Association between dietary vitamin C and sleep apnea in participants with and without hypertension. Table S6: Association between dietary vitamin C intake and insomnia. Table S7: Association between dietary vitamin C intake and insomnia after gender stratification. Table S8: Association between dietary vitamin C intake and insomnia after age stratification. Table S9: Association between dietary vitamin C and insomnia in participants with and without hypertension. Table S10: Association between dietary vitamin C intake and other types of sleep disorders. Table S11: Association between dietary vitamin C intake and other types of sleep disorders after gender stratification. Table S12: Association between dietary vitamin C intake and other types of sleep disorders after age stratification. Table S13: Association between dietary vitamin C and other types of sleep disorders in participants with and without hypertension. Figure S1: The results of dose–response between dietary vitamin C intake and sleep disorders in males. Figure S2: The results of dose–response between dietary vitamin C intake and sleep disorders in individuals aged ≤ 60. Figure S3: The results of dose–response between dietary vitamin C intake and sleep disorders in individuals with hypertension.
