# Supplementary material for: The Association Between Dietary Vitamin C and Sleep Disorders: A Cohort Study Based on UK Biobank
Source: Nutrients. 2024 Oct 28;16(21):3661. doi: 10.3390/nu16213661 (PMC11547431; doi:10.3390/nu16213661)

**Table S1.** Codes and names of sleep disorders defined according to ICD10.

| Diagnostic code (ICD–10) | Category |
|---|---|
| G47.0 | Disorders of initiating and maintaining sleep [insomnias] |
| G47.1 | Disorders of excessive somnolence [hypersomnias] |
| G47.2 | Disorders of the sleep–wake schedule |
| G47.3 | Sleep apnoea |
| G47.4 | Narcolepsy and cataplexy |
| G47.8 | Other sleep disorders |
| G47.9 | Sleep disorder, unspecified |

**Table S2.** Association between dietary vitamin C intake and sleep apnea.

| Model | Vitamin C intake(mg/d) | HR | Lower | Upper | *p*–Value |
|---|---|---|---|---|---|
| Model1 | Q1(<51.27) | | | Reference | |
| | Q2(51.27–90.44) | 0.733 | 0.610 | 0.881 | 0.001 |
| | Q3(90.44–132.88) | 0.819 | 0.685 | 0.979 | 0.028 |
| | Q4(132.88–191.51) | 0.608 | 0.500 | 0.738 | <0.001 |
| | Q5(>191.51) | 0.761 | 0.633 | 0.914 | 0.004 |
| Model2 | Q1(<51.27) | | | Reference | |
| | Q2(51.27–90.44) | 0.751 | 0.624 | 0.903 | 0.002 |
| | Q3(90.44–132.88) | 0.835 | 0.698 | 0.999 | 0.048 |
| | Q4(132.88–191.51) | 0.628 | 0.516 | 0.763 | <0.001 |
| | Q5(>191.51) | 0.794 | 0.661 | 0.955 | 0.014 |
| Model3 | Q1(<51.27) | | | Reference | |
| | Q2(51.27–90.44) | 0.830 | 0.689 | 1.000 | 0.050 |
| | Q3(90.44–132.88) | 0.953 | 0.795 | 1.143 | 0.603 |
| | Q4(132.88–191.51) | 0.752 | 0.616 | 0.918 | 0.005 |
| | Q5(>191.51) | 0.927 | 0.765 | 1.123 | 0.439 |

Model 1 serves as the crude model.

Model 2 adjusts for age and gender.

Model 3 incorporates additional variables, including education level, household income, Townsend Deprivation Index (TDI), ethnicity, BMI, physical activity, smoking status, alcohol drinker status, total energy intake, vitamin C supplements, hypertension, diabetes, stroke, and malignant neoplasms.

**Table S3.** Association between dietary vitamin C intake and sleep apnea after gender stratification.

| Gender | Vitamin C intake(mg/d) | HR | Lower | Upper | *p*–Value |
|---|---|---|---|---|---|
| Male | Q1(<51.27) | | Reference | | |
| | Q2(51.27–90.44) | 0.863 | 0.689 | 1.080 | 0.198 |
| | Q3(90.44–132.88) | 0.987 | 0.792 | 1.229 | 0.906 |
| | Q4(132.88–191.51) | 0.765 | 0.600 | 0.974 | 0.030 |
| | Q5(>191.51) | 0.886 | 0.699 | 1.124 | 0.320 |
| Female | Q1(<51.27) | | Reference | | |
| | Q2(51.27–90.44) | 0.775 | 0.556 | 1.081 | 0.133 |
| | Q3(90.44–132.88) | 0.889 | 0.643 | 1.229 | 0.477 |
| | Q4(132.88–191.51) | 0.728 | 0.511 | 1.035 | 0.077 |
| | Q5(>191.51) | 1.008 | 0.726 | 1.399 | 0.961 |

Covariates adjusted according to model 3.

**Table S4.** Association between dietary vitamin C intake and sleep apnea after age stratification.

| Age | Variable | HR | Lower | Upper | *p*–Value |
|---|---|---|---|---|---|
| ≤60 | Q1(<51.27) | | | Reference | |
| | Q2(51.27–90.44) | 0.764 | 0.604 | 0.967 | 0.025 |
| | Q3(90.44–132.88) | 0.896 | 0.711 | 1.128 | 0.348 |
| | Q4(132.88–191.51) | 0.671 | 0.516 | 0.872 | 0.003 |
| | Q5(>191.51) | 0.855 | 0.669 | 1.094 | 0.212 |
| >60 | Q1(<51.27) | | | Reference | |
| | Q2(51.27–90.44) | 0.972 | 0.713 | 1.327 | 0.859 |
| | Q3(90.44–132.88) | 1.081 | 0.800 | 1.462 | 0.612 |
| | Q4(132.88–191.51) | 0.895 | 0.650 | 1.232 | 0.496 |
| | Q5(>191.51) | 1.057 | 0.771 | 1.449 | 0.729 |

Covariates adjusted according to model 3.

**Table S5.** Association between dietary vitamin C and sleep apnoea in participants with and without hypertension.

| Outcome | Vitamin C intake (mg/d) | HR | Lower | Upper | *p*-Value |
|---|---|---|---|---|---|
| Hypertension | Q1(<51.27) | | Reference | | |
| | Q2(51.27–90.44) | 0.867 | 0.691 | 1.086 | 0.214 |
| | Q3(90.44–132.88) | 0.980 | 0.785 | 1.223 | 0.856 |
| | Q4(132.88–191.51) | 0.751 | 0.587 | 0.961 | 0.023 |
| | Q5(>191.51) | 0.925 | 0.732 | 1.170 | 0.517 |
| Non–hypertension | Q1(<51.27) | | Reference | | |
| | Q2(51.27–90.44) | 0.765 | 0.550 | 1.064 | 0.111 |
| | Q3(90.44–132.88) | 0.910 | 0.663 | 1.249 | 0.561 |
| | Q4(132.88–191.51) | 0.770 | 0.548 | 1.082 | 0.132 |
| | Q5(>191.51) | 0.941 | 0.673 | 1.315 | 0.721 |

Covariates adjusted according to model 3.

**Table S6.** Association between dietary vitamin C intake and insomnia.

| Model | Vitamin C intake (mg/d) | HR | Lower | Upper | *p*-Value |
|---|---|---|---|---|---|
| Model1 | Q1(<51.27) | Reference | | | |
| | Q2(51.27–90.44) | 0.941 | 0.531 | 1.668 | 0.836 |
| | Q3(90.44–132.88) | 0.733 | 0.398 | 1.351 | 0.320 |
| | Q4(132.88–191.51) | 0.943 | 0.532 | 1.670 | 0.840 |
| | Q5(>191.51) | 1.008 | 0.572 | 1.775 | 0.978 |
| Model2 | Q1(<51.27) | 1.000 | | | |
| | Q2(51.27–90.44) | 0.888 | 0.501 | 1.574 | 0.684 |
| | Q3(90.44–132.88) | 0.676 | 0.366 | 1.248 | 0.210 |
| | Q4(132.88–191.51) | 0.860 | 0.485 | 1.528 | 0.608 |
| | Q5(>191.51) | 0.913 | 0.517 | 1.613 | 0.755 |
| Model3 | Q1(<51.27) | Reference | | | |
| | Q2(51.27–90.44) | 0.896 | 0.503 | 1.595 | 0.709 |
| | Q3(90.44–132.88) | 0.682 | 0.367 | 1.266 | 0.225 |
| | Q4(132.88–191.51) | 0.876 | 0.487 | 1.575 | 0.658 |
| | Q5(>191.51) | 0.883 | 0.490 | 1.593 | 0.679 |

Model 1 serves as the crude model.
Model 2 adjusts for age and gender.
Model 3 incorporates additional variables, including education level, household income, Townsend Deprivation Index (TDI), ethnicity, BMI, physical activity, smoking status, alcohol drinker status, total energy intake, vitamin C supplements, hypertension, diabetes, stroke, and malignant neoplasms.

**Table S7.** Association between dietary vitamin C intake and insomnia after gender stratification.

| Gender | Vitamin C intake (mg/d) | HR | Lower | Upper | *p-V*value |
|---|---|---|---|---|---|
| Male | Q1(<51.27) | | Reference | | |
| | Q2(51.27–90.44) | 0.688 | 0.295 | 1.602 | 0.386 |
| | Q3(90.44–132.88) | 0.448 | 0.170 | 1.182 | 0.105 |
| | Q4(132.88–191.51) | 0.548 | 0.216 | 1.391 | 0.206 |
| | Q5(>191.51) | 0.391 | 0.136 | 1.123 | 0.081 |
| Female | Q1(<51.27) | | Reference | | |
| | Q2(51.27–90.44) | 1.209 | 0.534 | 2.736 | 0.649 |
| | Q3(90.44–132.88) | 1.008 | 0.432 | 2.356 | 0.985 |
| | Q4(132.88–191.51) | 1.319 | 0.587 | 2.962 | 0.503 |
| | Q5(>191.51) | 1.474 | 0.665 | 3.270 | 0.339 |

Covariates adjusted according to model 3.

**Table S8.** Association between dietary vitamin C intake and insomnia after age stratification.

| Age | Vitamin C intake (mg/d) | HR | Lower | Upper | *p-V*alue |
|---|---|---|---|---|---|
| ≤60 | Q1(<51.27) | | | Reference | |
| | Q2(51.27–90.44) | 1.044 | 0.480 | 2.269 | 0.914 |
| | Q3(90.44–132.88) | 0.857 | 0.372 | 1.976 | 0.717 |
| | Q4(132.88–191.51) | 0.896 | 0.385 | 2.085 | 0.799 |
| | Q5(>191.51) | 1.130 | 0.507 | 2.519 | 0.765 |
| > 60 | Q1(<51.27) | | | Reference | |
| | Q2(51.27–90.44) | 0.725 | 0.306 | 1.719 | 0.466 |
| | Q3(90.44–132.88) | 0.513 | 0.204 | 1.290 | 0.156 |
| | Q4(132.88–191.51) | 0.789 | 0.346 | 1.802 | 0.574 |
| | Q5(>191.51) | 0.636 | 0.266 | 1.522 | 0.310 |

Covariates adjusted according to model 3.

**Table S9.** Association between dietary vitamin C and insomnia in participants with and without hypertension.

| Outcome | Vitamin C intake (mg/d) | HR | Lower | Upper | p-Value |
|---|---|---|---|---|---|
| Hypertension | Q1(<51.27) | | Reference | | |
| | Q2(51.27–90.44) | 0.764 | 0.369 | 1.584 | 0.469 |
| | Q3(90.44–132.88) | 0.458 | 0.195 | 1.073 | 0.072 |
| | Q4(132.88–191.51) | 0.840 | 0.405 | 1.742 | 0.639 |
| | Q5(>191.51) | 0.672 | 0.311 | 1.452 | 0.313 |
| Non–hypertension | Q1(<51.27) | | Reference | | |
| | Q2(51.27–90.44) | 1.232 | 0.466 | 3.258 | 0.673 |
| | Q3(90.44–132.88) | 1.196 | 0.450 | 3.180 | 0.719 |
| | Q4(132.88–191.51) | 1.007 | 0.368 | 2.761 | 0.989 |
| | Q5(>191.51) | 1.333 | 0.506 | 3.509 | 0.561 |

Covariates adjusted according to model 3.

**Table S10.** Association between dietary vitamin C intake and other types of sleep disorders.

| Model | Vitamin C intake (mg/d) | HR | Lower | Upper | *p-V*value |
|---|---|---|---|---|---|
| Model1 | Q1(<51.27) | | | Reference | |
| | Q2(51.27–90.44) | 0.765 | 0.432 | 1.353 | 0.357 |
| | Q3(90.44–132.88) | 0.545 | 0.290 | 1.024 | 0.059 |
| | Q4(132.88–191.51) | 0.840 | 0.481 | 1.464 | 0.538 |
| | Q5(>191.51) | 0.973 | 0.568 | 1.667 | 0.921 |
| Model2 | Q1(<51.27) | | | Reference | |
| | Q2(51.27–90.44) | 0.790 | 0.446 | 1.398 | 0.418 |
| | Q3(90.44–132.88) | 0.565 | 0.300 | 1.064 | 0.077 |
| | Q4(132.88–191.51) | 0.880 | 0.503 | 1.538 | 0.654 |
| | Q5(>191.51) | 1.027 | 0.598 | 1.764 | 0.923 |
| Model3 | Q1(<51.27) | | | Reference | |
| | Q2(51.27–90.44) | 0.863 | 0.485 | 1.534 | 0.615 |
| | Q3(90.44–132.88) | 0.630 | 0.332 | 1.193 | 0.156 |
| | Q4(132.88–191.51) | 1.027 | 0.580 | 1.820 | 0.926 |
| | Q5(>191.51) | 1.177 | 0.670 | 2.067 | 0.572 |

Model 1 serves as the crude model.

Model 2 adjusts for age and gender.

Model 3 incorporates additional variables, including education level, household income, Townsend Deprivation Index (TDI), ethnicity, BMI, physical activity, smoking status, alcohol drinker status, total energy intake, vitamin C supplements, hypertension, diabetes, stroke, and malignant neoplasms.

**Table S11.** Association between dietary vitamin C intake and other types of sleep disorders after gender stratification.

| Gender | Vitamin C intake (mg/d) | HR | Lower | Upper | *p-V*alue |
|---|---|---|---|---|---|
| Male | Q1(<51.27) | | Reference | | |
| | Q2(51.27–90.44) | 0.965 | 0.468 | 1.991 | 0.924 |
| | Q3(90.44–132.88) | 0.553 | 0.235 | 1.305 | 0.176 |
| | Q4(132.88–191.51) | 0.960 | 0.454 | 2.032 | 0.916 |
| | Q5(>191.51) | 1.048 | 0.497 | 2.214 | 0.901 |
| Female | Q1(<51.27) | | Reference | | |
| | Q2(51.27–90.44) | 0.720 | 0.277 | 1.871 | 0.500 |
| | Q3(90.44–132.88) | 0.759 | 0.290 | 1.987 | 0.575 |
| | Q4(132.88–191.51) | 1.178 | 0.487 | 2.848 | 0.717 |
| | Q5(>191.51) | 1.421 | 0.602 | 3.353 | 0.423 |

Covariates adjusted according to model 3.

**Table S12.** Association between dietary vitamin C intake and other types of sleep disorders after age stratification.

| Age | Vitamin C intake (mg/d) | HR | Lower | Upper | *p-V*alue |
|---|---|---|---|---|---|
| ≤60 | Q1(<51.27) | | | Reference | |
| | Q2(51.27–90.44) | 0.603 | 0.290 | 1.251 | 0.174 |
| | Q3(90.44–132.88) | 0.588 | 0.276 | 1.254 | 0.169 |
| | Q4(132.88–191.51) | 0.960 | 0.489 | 1.887 | 0.906 |
| | Q5(>191.51) | 0.978 | 0.492 | 1.944 | 0.950 |
| > 60 | Q1(<51.27) | | | Reference | |
| | Q2(51.27–90.44) | 1.882 | 0.640 | 5.534 | 0.251 |
| | Q3(90.44–132.88) | 0.888 | 0.255 | 3.099 | 0.853 |
| | Q4(132.88–191.51) | 1.472 | 0.472 | 4.594 | 0.506 |
| | Q5(>191.51) | 1.986 | 0.667 | 5.914 | 0.218 |

Covariates adjusted according to model 3.

**Table S13.** Association between dietary vitamin C and other types of sleep disorders in participants with and without hypertension.

| Outcome | Vitamin C intake (mg/d) | HR | Lower | Upper | *p-V*alue |
|---|---|---|---|---|---|
| Hypertension | Q1(<51.27) | | Reference | | |
| | Q2(51.27–90.44) | 1.259 | 0.603 | 2.631 | 0.540 |
| | Q3(90.44–132.88) | 0.434 | 0.155 | 1.218 | 0.113 |
| | Q4(132.88–191.51) | 0.939 | 0.408 | 2.161 | 0.883 |
| | Q5(>191.51) | 1.247 | 0.573 | 2.715 | 0.578 |
| Non–hypertension | Q1(<51.27) | | Reference | | |
| | Q2(51.27–90.44) | 0.480 | 0.181 | 1.270 | 0.139 |
| | Q3(90.44–132.88) | 0.816 | 0.353 | 1.882 | 0.633 |
| | Q4(132.88–191.51) | 1.096 | 0.497 | 2.417 | 0.820 |
| | Q5(>191.51) | 1.099 | 0.484 | 2.496 | 0.822 |

Covariates adjusted according to model 3.

**Figure S1.** The results of dose–response between dietary vitamin C intake and sleep disorders in males.

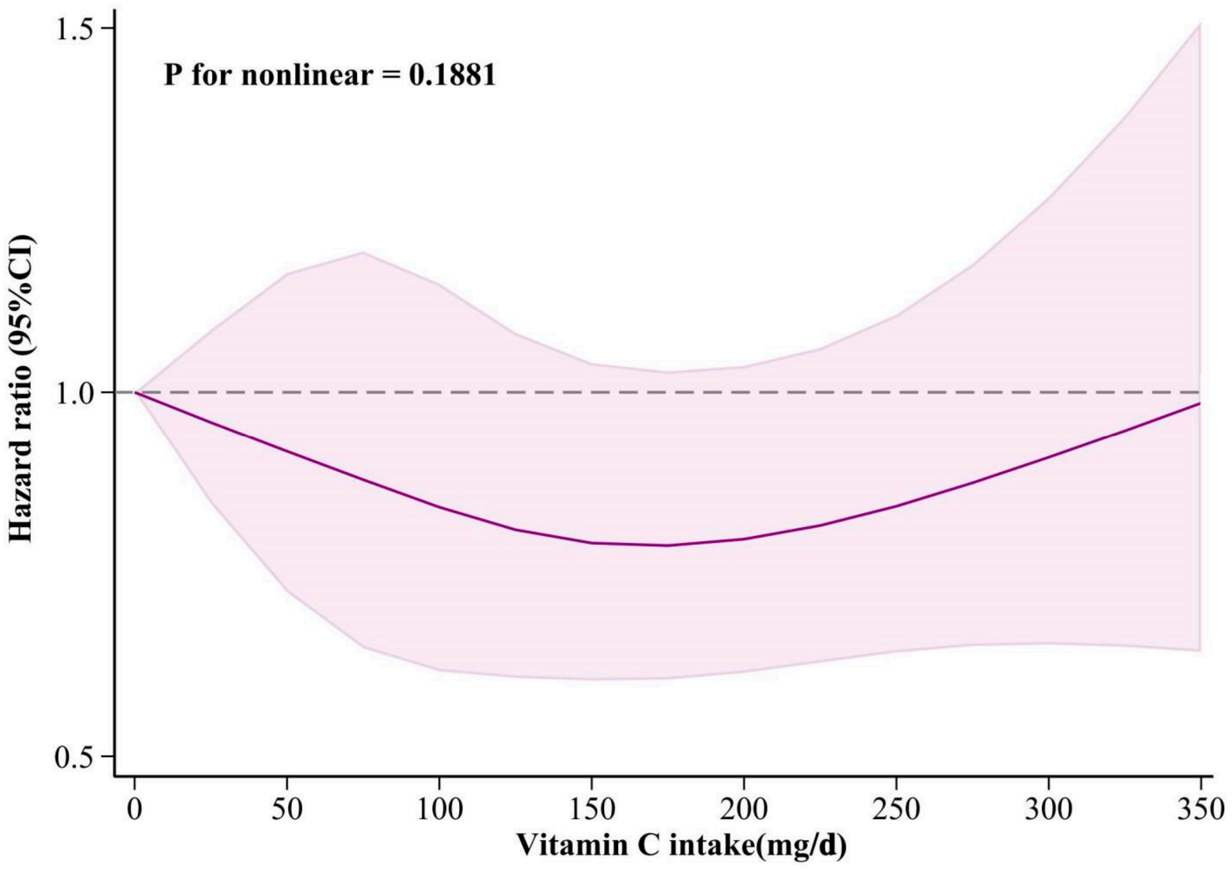

**Figure S2.** The results of dose–response between dietary vitamin C intake and sleep disorders in individuals aged ≤60.

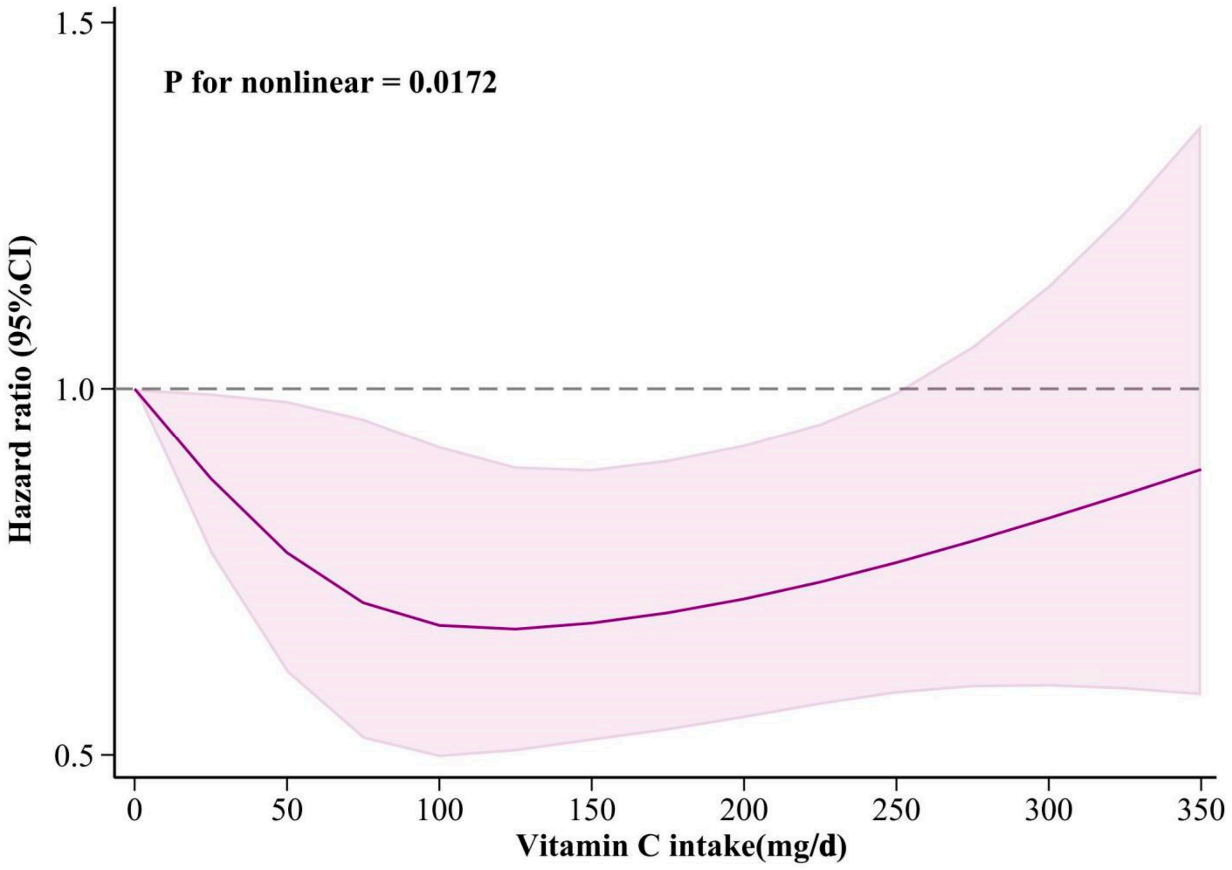

**Figure S3.** The results of dose–response between dietary vitamin C intake and sleep disorders in individuals with hypertension.

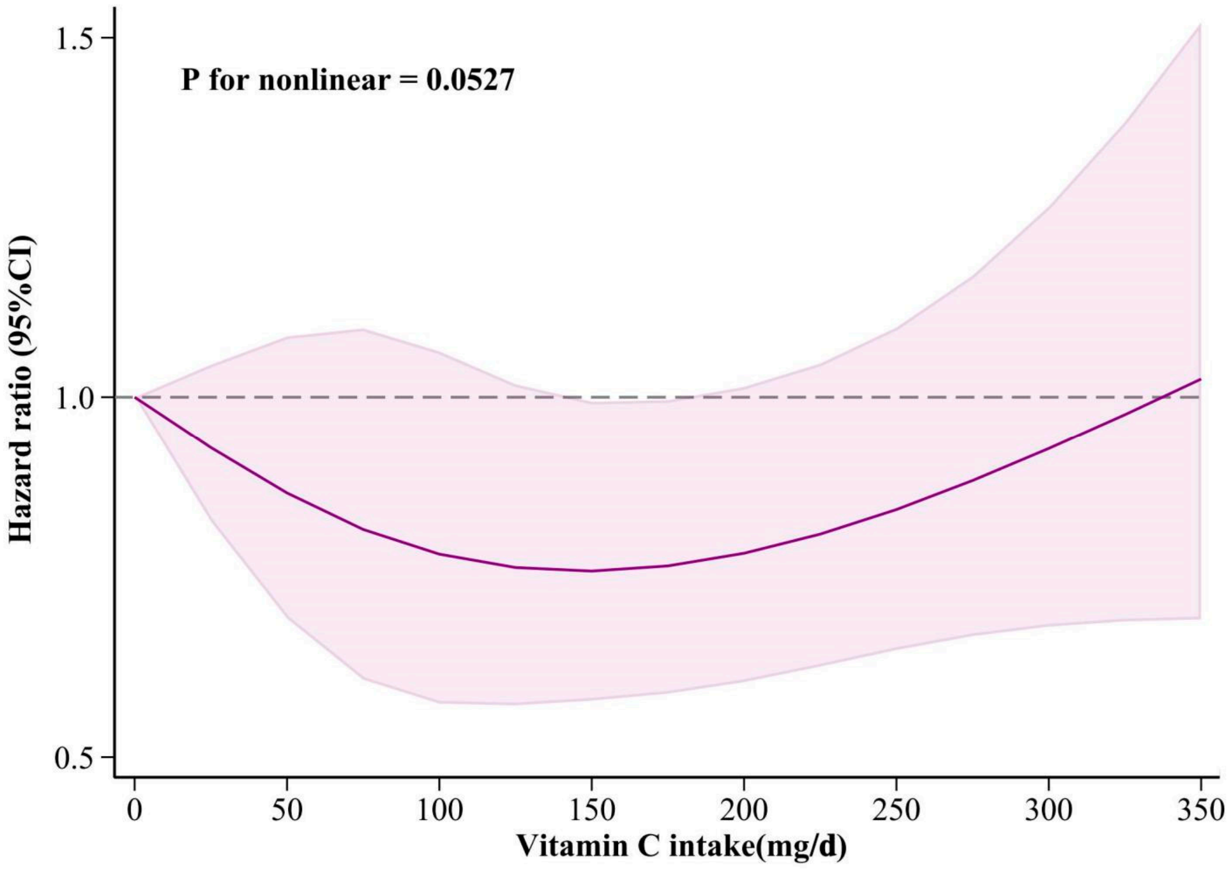

Supplement: Supplementary file 1 [file nutrients-16-03661-s001.zip › nutrients-3222410-supplementary.pdf]
